# Primary splenic T-cell/histiocyte-rich B-cell lymphoma in a patient with recurrent hairy cell leukemia: a case report

**DOI:** 10.1093/omcr/omac123

**Published:** 2022-11-24

**Authors:** Tagrid Younes Ahmad, Hasan Nabil Al Houri, Sami Jomaa, Wisam Assad, Sarah Zaher Addeen

**Affiliations:** Neurology Department, Tishreen Military Hospital, Damascus, Syria; Faculty of Medicine, Syrian Private University, Damascus, Syria; Faculty of Medicine, Syrian Private University, Damascus, Syria; Internal Medicine Department, Damascus University, Damascus, Syria; Faculty of Medicine, Damascus University, Damascus, Syria; Faculty of Medicine, Syrian Private University, Damascus, Syria; Pathology Department, Al Mouwasat University Hospital, Damascus, Syria; Ophthalmology Department, Al Mouwasat University Hospital, Damascus University, Damascus, Syria

## Abstract

T-cell/histiocyte-rich B-cell lymphoma is a high-grade, morphologic variant of diffuse large B-cell lymphoma. T-cell/histiocyte-rich B-cell lymphoma. It is rare as a primary splenic involvement and is usually reported as a second malignancy after hairy cell leukemia. Here, we report the first case that describes the occurrence of primary splenic T-cell/histiocyte-rich B-cell lymphoma in a patient with a previous diagnosis of recurrent hairy cell leukemia. A 53-year-old male patient was diagnosed with hairy cell leukemia in 1996 and achieved complete remission with Pentostatin. Then, recurrence of hairy cell leukemia was diagnosed in 2015 and treated with Cladribine. In 2016, he presented with B symptoms and hypersplenism. Therapeutic and diagnostic splenectomy was performed. Histopathological study with immunohistochemistry evaluation revealed the presence of T-cell/histiocyte-rich B-cell lymphoma. Therefore, second malignancies should be considered in patients with a previous neoplasm when symptoms recur or develop.

## BACKGROUND

Hairy cell leukemia (HCL) is a chronic, low-grade malignant lymphoproliferative disorder that accounts for 2% of all leukemia cases [1–3]. It is characterized by the accumulation of small, mature B-cells with abundant cytoplasm and hair-like cytoplasmic projections, with or without histiocytes within peripheral blood, bone marrow, liver and splenic red pulp [1, 4]. The incidence of second malignancies has increased in patients with hairy cell leukemia, either due to the disease itself or secondary to the immunosuppressive effects of the therapy [5]. T-cell/histiocyte-rich B-cell lymphoma (THRLBCL) is a high-grade, morphologic variant of diffuse large B-cell lymphoma (DLBCL). It constitutes 2–3% of all B-cell lymphomas [1, 6]. The primary occurrence of this subtype in the spleen is scarce. However, up to our knowledge, this is the first case that describes the development of primary splenic THRLBCL in a patient who was previously diagnosed with recurrent HCL.

## CASE PRESENTATION

**Figure 1:**
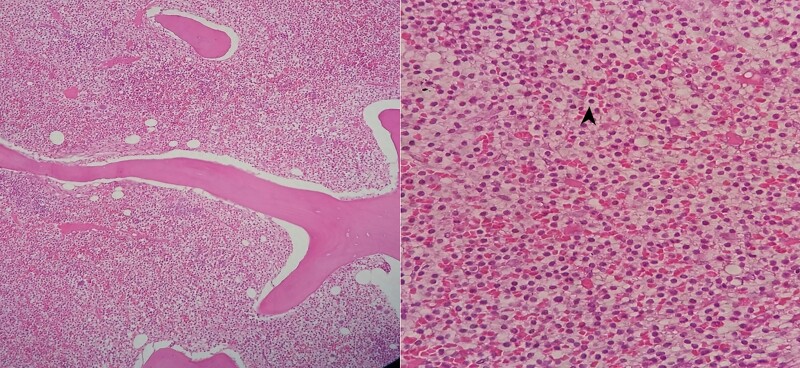
The bone marrow biopsy sections reveal infiltration by a group of large cells contains clear to eosinophilic cytoplasm and perinuclear halo (arrow head). Nuclei with variable chromatin, rare mitosis figures and focal bone marrow fibrosis were observed. No prominent nucleoli were noticed (×100).

**Figure 2:**
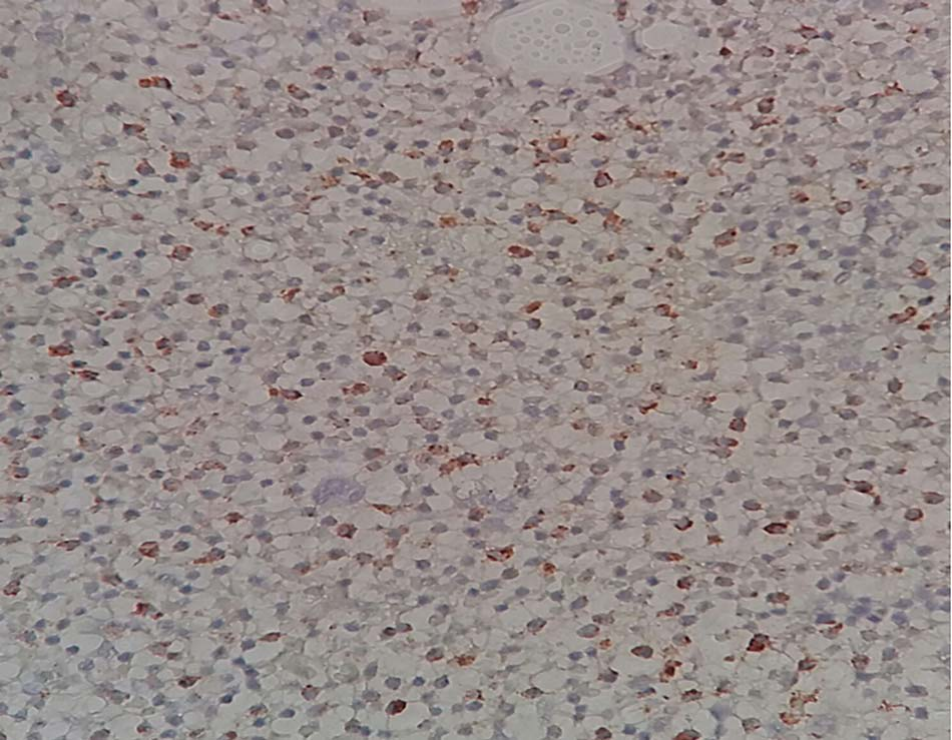
The section of bone marrow shows positivity of Hairy cell antibody on large cells (Anti-Hairy Antibodies (1/24)).

A 53-year-old male patient was diagnosed with hairy cell leukemia (HCL) in 1996 following symptoms of fatigue, recurrent infections and easy bruising. Complete remission was achieved with Pentostatin. In 2015, he developed malaise, hepatomegaly and splenomegaly. Bone marrow biopsy revealed hypercellular bone marrow (75%), with focal infiltration by groups of large cells with clear eosinophilic cytoplasm. Nuclei with perinuclear halos contain variable chromatin, and rare mitosis figures without prominent nucleoli were observed. There was a moderate increase in reticulin fibers (Fig. 1). A mouse monoclonal IgG1 κ Hairy for the Dako immunohistochemistry was used. Bone marrow immunohistochemistry staining was positive for CD20 and hairy cell antibodies [Anti-Hairy Antibodies (1/24)] on large cells (Fig. 2). Myeloperoxidase (MPO) was negative. The histopathological picture and immunohistochemistry findings confirmed the relapse of HCL. We achieved complete remission with Cladribine. The patient continued to do well until June 2016, when he was admitted to our hospital with a history of B-symptoms. Vital signs were within normal range. Clinical examination revealed grade I in-depth and II in-extension lower limbs with edema and a palpable spleen, 12 cm below the costal edge. The patient could not afford the high cost of flow cytometry, so we used antibody testing to confirm the diagnosis.

The patient was presumed to have a new relapse of HCL as the new bone marrow biopsy was normal. In addition, we found no plasma cells on bone marrow biopsy, which excludes the blastic transformation of hairy cell leukemia. An abdominal ultrasound showed a massive homogenous splenomegaly (26 × 16) cm. Laboratory tests revealed pancytopenia with an increased erythrocyte sedimentation rate (ESR) and lactate dehydrogenase (LDH). Peripheral blood smear exhibited some anisocytosis with a few tear-shaped red cells and a significant decrease in white blood cells (WBCs) and platelet counts. Echocardiography exposed preserved Ejection Fraction (EF) and mild regurgitation in the mitral, tricuspid and pulmonary valves with no significant gradient. Electrocardiogram (ECG) and chest X-ray (CXR) were within normal. Abdominal computed tomography (CT) scan confirmed homogenous splenomegaly (26.3× 16) cm, without other abnormalities. We transferred the patient to perform a diagnostic and therapeutic splenectomy due to suspicion of hypersplenism and without a definitive diagnosis.

**Figure 3:**
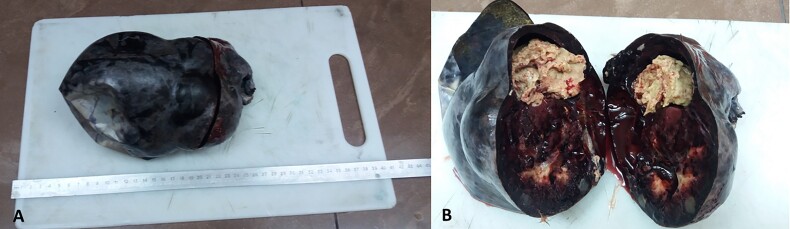
A) gross examination reveals a huge splenomegaly. B) Gross examination of two splenic segments measuring (24× 9× 8) cm and (22× 9× 8.5) cm.

**Figure 4:**
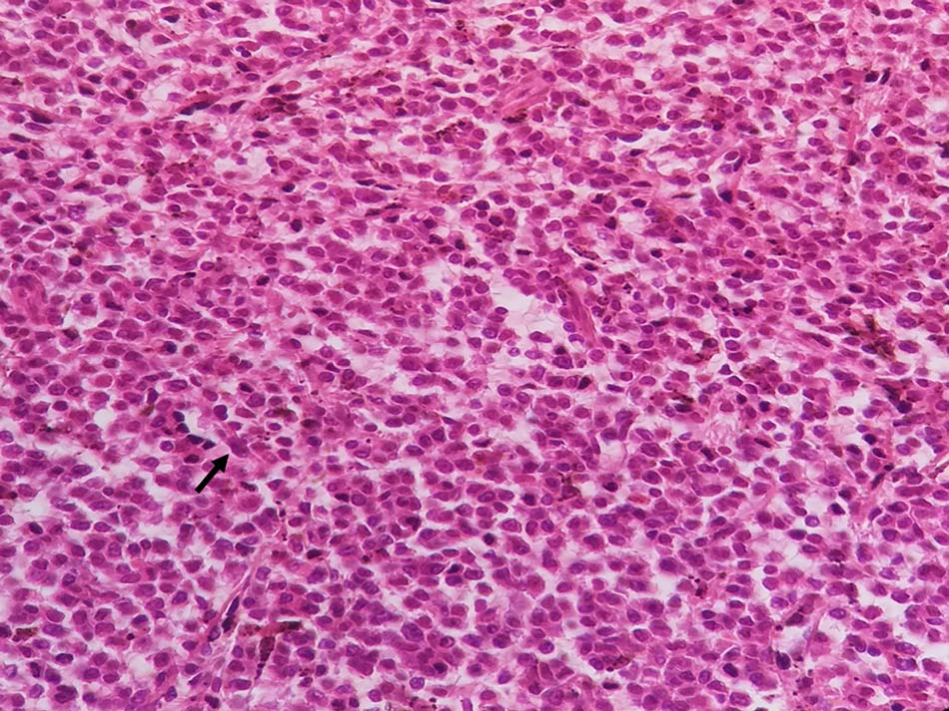
Microscopic examination on spleen specimen using hematoxylin and eosin staining reveals spleen tissue with infiltrations of monoclonal large lymphocytes with abundant cytoplasm and eccentric nuclei (black arrow).

**Figure 5:**
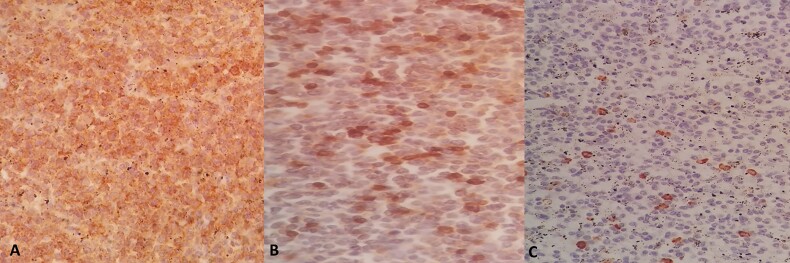
A) Immunohistochemistry evaluation of spleen specimen shows positivity of CD20 on large cells. B) Immunohistochemistry evaluation of spleen specimen shows positivity of CD3 on some of surrounding small lymphocytes. C) Immunohistochemistry evaluation of spleen specimen shows positivity of CD15.

**Figure 6:**
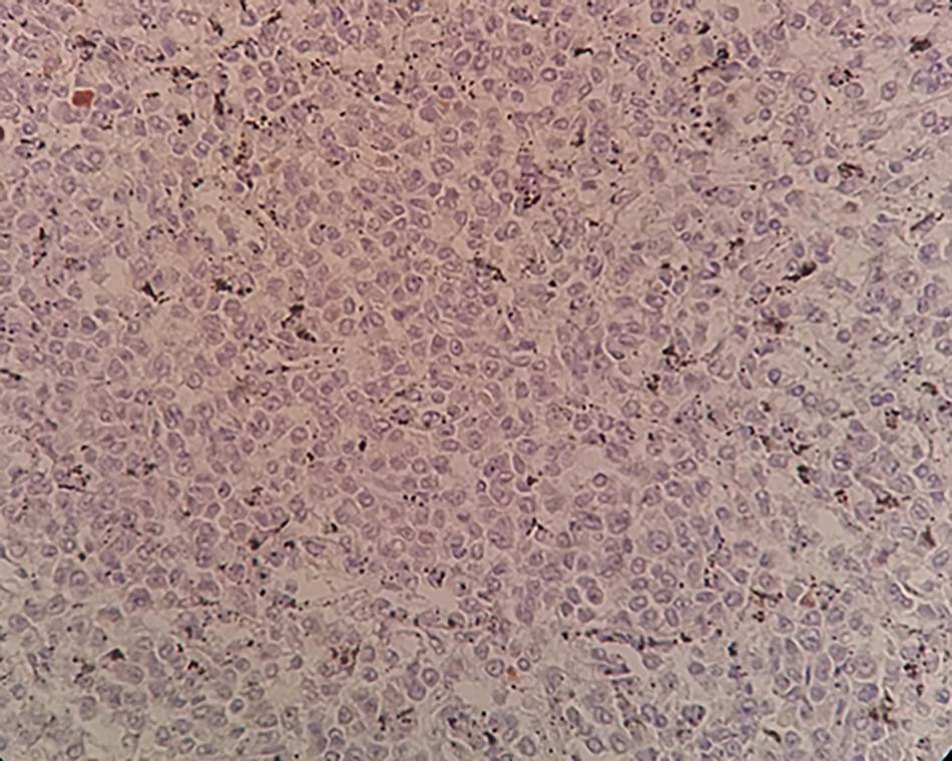
A negative Annexin A1 staining on tumor cells within spleen tissue.

**Figure 7:**
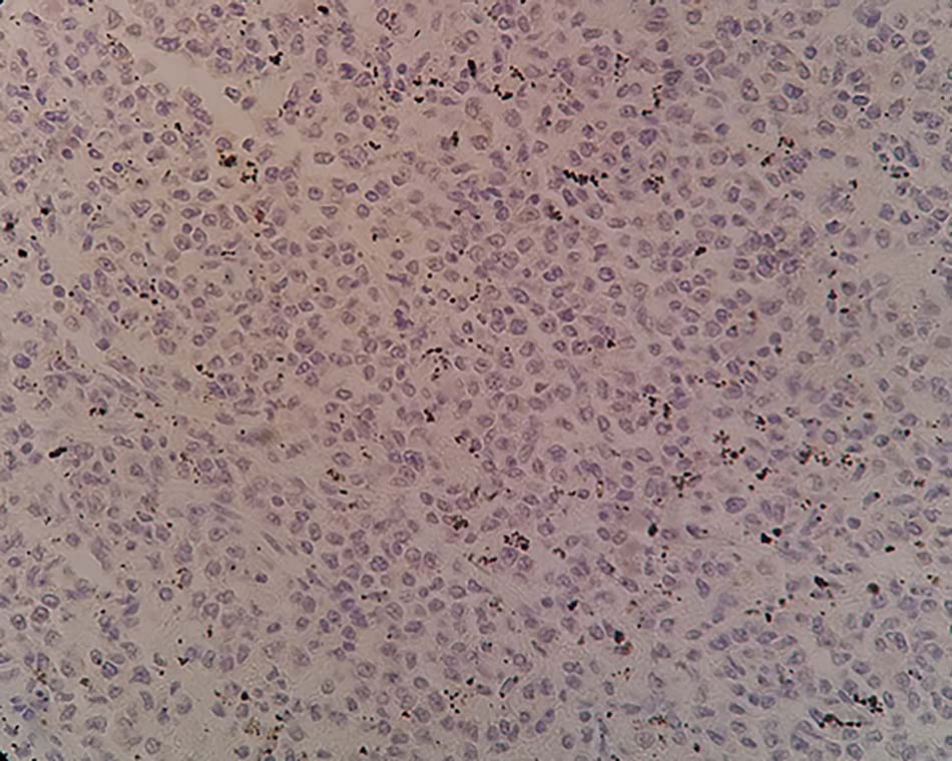
A negative TRAP staining on tumor cells within spleen tissue.

**Figure 8:**
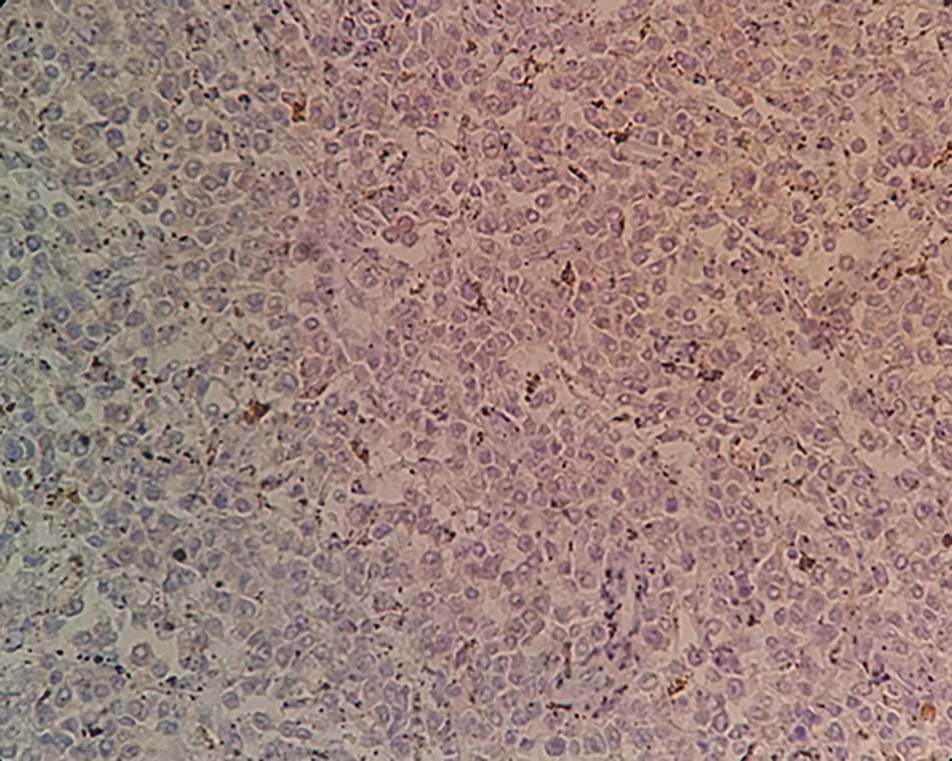
A negative CD103 staining on tumor cells within spleen tissue.

Gross examination showed massive splenomegaly (Fig. 3A). The spleen was cut into two segments measuring (24× 9× 8) cm and (22× 9× 8.5) cm (Fig. 3B). Microscopic examination using hematoxylin and eosin (HE) revealed splenic tissue infiltrated by monoclonal, large lymphocytes with abundant cytoplasm and eccentric nuclei (Fig. 4). Immunohistochemistry evaluation results were positive for CD20 on large cells (Fig. 5A), CD3 on some of the background small lymphocytes (Fig. 5B), CD15 (Fig. 5C) and CD45. However, it was negative for CD30 and hairy cell staining. To exclude the possibility of blastic transformation of hairy cell leukemia, we performed additional staining for Annexin A1 (Fig. 6), TRAP (Fig. 7) and CD103 (Fig. 8) in a private histology laboratory, which all became negative. Staining for CD22 and CD25 was not available in our country; therefore, we made the final diagnosis based on the positivity of CD3, CD15, CD20 and CD45 and the negativity of Annexin A1, TRAP and CD103. These results confirmed the diagnosis of T-cell rich B cell lymphoma. The patient was referred to the hematology-oncology department to receive chemotherapy. The patient received six cycles of R-CHOP. The patient had an uneventful follow-up until Feb 2021, when we lost the follow-up with him for unknown reasons.

## DISCUSSION

Hairy cell leukemia (HCL) is a rare type of low-grade non-Hodgkin lymphoma (NHL), which is considered a hematological distinct of chronic leukemia, accounting for about 2% of all leukemias [1, 2]. HCL typically occurs in middle-aged men presenting with pancytopenia and marked susceptibility to infection. Morphologically, it is characterized by typical hairy cells with irregular, tartrate-resistant acid phosphatase (TRAP) positive cytoplasmic projections [7–10]. HCL, hairy cell leukemia—variant (HCL-v) and splenic diffuse red pulp small B-cell lymphoma may have cytoplasmic projections. Therefore, it is crucial to differentiate HCL from other diseases [10]. HCL expresses TRAP and stains positive for annexin A1, cyclin D1, CD11c, CD25, CD103 and CD123. HCL also negatively stains CD5, CD23 and CD10 [9, 10]. Although HCL-v could be TRAP-positive, CD25, cyclin D1 and annexin A1 are characteristically absent. Splenic diffuse red pulp small B-cell lymphoma (previously diagnosed as splenic lymphoma with villous lymphocytes) lacks annexin A1, CD5, CD25, CD103 and CD11c [10]. To exclude the blastic transformation of HCL in our case, we performed staining Annexin A1, TRAP and CD103, which all became negative. However, staining for CD22 and CD25 was not

available in our country; therefore, we made the final diagnosis based on the positivity of CD3, CD15, CD20 and CD45 and the negativity of Annexin A1, TRAP and CD103.

T-cell/histiocyte-rich B-cell Lymphoma (THRLBCL) is a new clinicopathological entity. It was first described in 1988. It constitutes almost 0.1% of all lymphomas. It is rare to encounter THRLBCL as a primary lymphoma in the spleen [1, 2]. The WHO classification of lymphomas included THRLBCL as a specific histologic subtype of DLBCL; and reserved its diagnosis for cases in which the large B-cell component accounts for 10% or less of tumor cells, surrounded by a majority population of small polyclonal T cells, with or without histiocytes [1]. The available knowledge about THRLBCL is scarce and mainly derived from published cases and case series. This subtype often has aggressive clinical behavior. Therefore, patients often present in advanced stages with splenomegaly and bone marrow involvement. Immunohistochemical staining for B-cells is always positive for CD20 and CD45, whereas there is a rarity of the positivity of CD30. More than 50% of cases are positive for BCL-2 [11]. The occurrence of a second malignancy with HCL synchronously or subsequently has been reported [12]. The most extensive study on this topic reported second cancer among 3104 patients with HCL and concluded that patients with HCL are at increased risk of Hodgkin lymphoma, non-Hodgkin lymphoma, leukemia and thyroid cancer [12].

Many theories were suggested to explain this phenomenon, the shared genetic predisposition, environmental exposure to carcinogens and immunosuppression [13]. Pentostatin or cladribine remains the standard first-line treatment of HCL and could achieve cure rates of 70–90%. However, a complete cure with purine analog therapy is uncertain as patients are still at risk for relapse after an average of 15–16 years [7]. On the other hand, Rituximab therapy showed cure rates of 10–54%, while a combination of purine analog and Rituximab could achieve cure rates of 88–100% [7, 14]. However, the latter is associated with increased immunosuppression, toxicities and second malignancies from purine analogs [7, 13]. Moreover, the splenectomy could worsen immunity and allow for mutations and subsequent transformation of HCL into a more severe neoplasm [15].

The patient, in this case, has undergone diagnostic/therapeutic splenectomy due to splenic recurrence of HCL. Splenectomy is a well-described part of HCL treatment that helps to enhance hematologic parameters [8, 9]. Surprisingly, the biopsy revealed the presence of THRLBCL as the malignancy in the spleen. Moreover, the flow cytometry came TRAP negative and HCL staining negative. However, it is difficult to make a definitive statement about whether the presence of TRCBCL was a coincidence or whether there is a causal relationship between the two malignancies. In our case, we rely on the negativity of Annexin A1, TRAP and CD103 to exclude the blastic transformation of HCL.

In conclusion, hairy cell leukemia is an indolent, chronic lymphoproliferative malignancy. It is common to develop a second malignancy in this leukemia, attributed to several theories. However, THRLBCL is rare as primary splenic involvement and a second malignancy after HCL. Second malignancies should be considered, whether symptoms relapsed or new ones developed in patients who previously had neoplasm, especially HCL. However, we recommend close monitoring for early detection of possible new malignancies in HCL patients.
